# Preparation and Evaluation of Polymer-Based Ultrasound Gel and Its Application in Ultrasonography

**DOI:** 10.3390/gels8010042

**Published:** 2022-01-06

**Authors:** Sadia Afzal, Muhammad Zahid, Zulfiqar Ahmad Rehan, H. M. Fayzan Shakir, Hamza Javed, Meshari M. H. Aljohani, Syed Khalid Mustafa, Maqsood Ahmad, Montaser M. Hassan

**Affiliations:** 1 Department of Chemistry, University of Agriculture, Faisalabad 38040, Pakistan; sadiaafzal1408@gmail.com (S.A.); zahid595@gmail.com (M.Z.); 2 Department of Materials, School of Engineering and Technology, National Textile University, Faisalabad 38040, Pakistan; 3 School of Materials Science and Engineering, Northwestern Polytechnical University, Xi’an 710072, China; 4 Multan Medical and Dental College, University of Health Sciences, Lahore 54600, Pakistan; hamzambbs96@gmail.com; 5 Department of Chemistry, Faculty of Science, University of Tabuk, Tabuk 71491, Saudi Arabia; mualjohani@ut.edu.sa (M.M.H.A.); khalid.mustafa938@gmail.com (S.K.M.); 6 Department of Agronomy, University of Agriculture Faisalabad, Faisalabad 38040, Pakistan; maqsood2043@gmail.com; 7 Department of Biology, College of Science, Taif University, P.O. Box 11099, Taif 21944, Saudi Arabia; m.sayd@tu.edu.sa

**Keywords:** ultrasonography, ultrasound gel, aloe vera, CAR 980, methylparaben

## Abstract

Ultrasound imaging is a widely used technique in every health care center and hospital. Ultrasound gel is used as a coupling medium in all ultrasound procedures to replace air between the transducer and the patient’s skin, as ultrasound waves have trouble in traveling through air. This research was performed to formulate an inexpensive alternative to commercially available ultrasound gel as it is expensive and imported from other countries. Different formulations with different concentrations of carbopol 980 (CAR 980) and methylparaben were prepared with natural ingredients such as aloe vera gel and certain available chemicals that have no harmful effects on the skin. To justify the efficiency of the formulations; necessary physicochemical characteristics such as visual clarity, homogeneity, transparency, skin irritation, antibacterial activity, pH, stability, spreadability, conductivity, acoustic impedance, viscosity, and cost were evaluated. Moreover, a comparison study was also conducted with commercially available ultrasound gel that was utilized as a control. All samples showed excellent transparency and no microbial growth. S1 was the only formulation that met all of the requirements for commercial ultrasound gel and produced images that were similar to those produced by commercial ultrasound gel. So, this formulation could be used as an alternative to expensive commercial ultrasound gel for taking images in hospitals and medical centers.

## 1. Introduction

Ultrasonography (ultrasound imaging) is an ultrasound-based medical imaging technique that is utilized for mapping or identifying internal aspects of the patient’s body, such as the fetus, muscles, and tendons [1,2,3,4]. Medical ultrasound imaging has advantages over magnetic resonance imaging (MRI), such as portability, real-time imaging, reasonable cost, and its harmless effect. However, the resolution of MRI systems and CT (computerized tomography) is usually higher than that of the ultrasound imaging system [5,6]. Ultrasound diagnostic procedures are used in assessing and diagnosing a wide variety of medical conditions related to internal organs in a non-invasive manner [2,7,8]. Ultrasound procedures are also utilized for treating skin conditions such as reducing wrinkles and treatment of warts [2,7,9].

These ultrasound procedures utilize ultrasound waves (sound waves of a frequency greater than 20 kHz, which is the upper limit of human hearing) and use a range of frequencies (1.5–20 MHz) depending on their applications [10,11]. These ultrasound procedures need a special medium that can provide lubrication to the skin to aid the movement of the transducer on the skin and can replace air between the transducer and the patient’s skin because ultrasound waves have difficulty in traveling through the air due to very small acoustic impedance (0.004 MRayls) [12,13,14,15]. Ultrasound gel is the best medium to achieve these functions. It transfers ultrasound waves between the patient’s skin and the transducer and reduces the mismatch of acoustic impedance as it has matching acoustic impedance with soft tissues (1.5 MRayls) for complete transmission of ultrasound waves. Complete transmission of waves results in good images [15,16,17,18,19,20].

In resource-limited settings, portable ultrasound is getting to be a progressively beneficial diagnostic device. Generally, current costs for ultrasound are low but the approach to the essential consumable goods (specifically ultrasound gel) is one of the major implementation hurdles. In resource-limited sites, the high cost and unreliable access to commercial ultrasound gel can limit its extensive and routine use [17,18,21]. Unluckily, certain chemicals were found to be used for the formulation of ultrasound gel that caused allergic reactions to the skin, such as isothiazolinones, methylisothiazolinone, phenoxyethanol (as preservatives), and Carbopol 940 (as a thickening agent), which are used in several cosmetics and industrial products. There are numerous publications describing patients who suffered from allergic dermatitis due to using these compounds in the formulation of the gel [22,23,24,25]. Furthermore, different types of polysaccharides were used in ultrasound gel formulations, which had some drawbacks such as clarity, transparency, homogeneity, and imaging quality [18,26,27,28,29].

**Figure 1:**
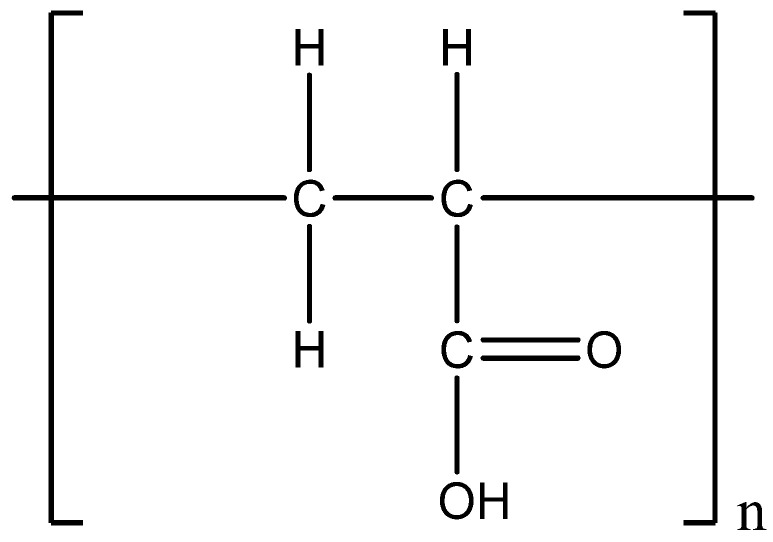
Chemical structure of a monomeric unit of acrylic acid in a carbomer polymer.

Aloe vera has anti-inflammatory, antimicrobial, antioxidant, antifungal, and anti-aging characteristics and promotes the healing of wounds. It also prevents the formation of stretch marks [30]. Aloe vera has synergistic activity due to its 75 active ingredients [13]. Ultrasound gels are prepared using fresh aloe vera gel, which does not irritate the skin and makes them safe for people with sensitive skin. Glycerine has become widely used as a skin-conditioning agent in a variety of pharmaceutical formulations and cosmetics. It is generally considered a relatively noncorrosive and nontoxic material [2,31]. Carbomers are high molecular weight synthetic polymers of acrylic acid that are cross-linked with polyalcohols or alkenyl ethers of sugars. Based on the level of cross-linking and mode of synthesis, various carbomer grades exist in the market, for example, Carbopol (CAR) 980, CAR 934, and CAR 940 [32,33]. When carbopol polymer is subjected to a pH range of 4.0 to 6.0, it swells 1000 times more than its initial volume to produce a gel. So, a neutralizing reagent like triethanolamine is used to jellify CAR molecules in various liquids [33,34,35]. The monomer unit of the carbomer is displayed in Figure 1.

In this research, an inexpensive, optimized, and good quality ultrasound gel was formulated by utilizing carbopol 980 (a synthetic polyacrylic acid polymer as a thickening agent), methylparaben and propylparaben (as a preservative), aloe vera gel (as an anti-inflammatory agent), glycerine (as a skin-conditioning agent), disodium EDTA (as a chelating agent), distilled water (as a vehicle), and TEA (as a neutralizing agent). The effect of synthesis parameters on the properties of ultrasound gel formulation was also examined like the effect of concentration of thickening agent on the viscosity of gel was examined by measuring the viscosity of gel with a rheometer. Its antimicrobial activity, skin irritancy, and quality of images produced were also evaluated along with a comparison study to commercial ultrasound gel.

## 2. Characterization

The physicochemical properties of all prepared formulations were evaluated by comparing the results of the prepared formulations to the results of a commercial ultrasound gel available in the market (commercial gel = CG), which was utilized as a control. To evaluate the prepared formulations, the following factors were utilized:

### 2.1. Visual Inspection

One of the most significant characteristics of ultrasound gels is clarity. The clarity of all prepared formulations was inspected visually against a white and black background [36,37]. Other physicochemical properties such as appearance, transparency, and color were also evaluated by visual examination.

### 2.2. Homogeneity

The homogeneity of all formulations was evaluated through visual examination of the prepared gel samples after they had settled into beakers properly and by touch. Gels were examined for the presence of any aggregates, their appearance, type of smear, how the gel was removed, and after-feel [11].

### 2.3. UV-Vis-NIR Spectrophotometry

The transmittance of all formulations, along with commercial gel (CG), was determined by the spectrophotometer (UV/V is /NIR spectrophotometer, Perkin Elmer, Boston USA, model no. Lambda 950, range: 150 to 3300 nm) in the visible range (400 to 600 nm).

### 2.4. pH Determination

pH is the most vital factor because of the three zones of crucial significance that are listed below: The impact of pH on skin, stability, and solubility. At the same time, any ultrasonic gel composition should have a pH that does not irritate the patient and ensure the formulation’s stability [11]. Triethanolamine was added drop wise with constant stirring to adjust the pH of prepared formulations and was measured by the pH paper frequently.

### 2.5. Skin Irritation Test

All formulations were put through a skin irritation analysis on human volunteers to see if any irritation issues would render them inappropriate for use. One gram of the gel sample was applied topically to a two square inch area of the hand. Observations were taken and recorded for any irritation, lesions, redness, or edema at periodic intervals for roughly 24 h [11,13].

### 2.6. Viscosity Determination

The viscosity of commercial gel and prepared formulations were measured to choose the one formulation among all that had the best matching viscosity with commercial gel. All measurements were taken by the rheometer (TA instruments, Model no. AR 1500 ex) and all experiments were performed at a 30 °C temperature. The sample was placed on the peltier plate or stationary plate whose temperature was set to 30 °C before starting the procedure. The measuring geometry used was a plate with a 40 mm diameter and a 2 mm gap. Then geometry was placed on the sample and started rotating from a minimum (0.05 rad/s) to a maximum (200 rad/s) angular frequency. Shear rate (1/s) was calculated from angular frequency by Equation (1) and dynamic viscosity (in Pa.s) was calculated by Equation (2) [38,39].

(1) $$\mathrm{Shear}\;\mathrm{rate}=\frac{r}{h}\times \Omega$$

(2) $$\mathrm{Dynamic}\;\mathrm{viscosity}=\frac{\mathrm{Shear}\;\mathrm{stress}}{\mathrm{shear}\;\mathrm{rate}}$$

where,

(3) $$\mathrm{shear}\;\mathrm{stress}=\frac{2}{{\pi r}^{3}}\times M$$

*r* = Radius of the plate geometry (m).

*h* = Gap between the plates (m).

M = Torque (Nm).

*Ω* = Motor angular velocity (rad/s).

### 2.7. Antibacterial Activity

The antibacterial activity of all formulations (S1, S2, … S9) along with a blank sample (S0), which contains no parabens at all, and commercial gel (CG), was analyzed against a Gram-negative bacteria (*Escherichia coli* or *E. coli*) by using the zone inhibition method. The prepared gel formulations and commercial gel as reference was inoculated on the plates of agar media by the well diffusion method. Then the plates were incubated for 24 h at 37 °C. Plates were removed after the incubation period and bacterial growth was examined by measuring the zone of inhibition using a zone reader in millimeters [13,40].

### 2.8. Conductivity

The conductivity values of ultrasound gel formulations were measured by utilizing the conductivity meter (BANTE INSTRUMENT, Conductivity/TDS/Salinity meter, Model No. 950, Shanghai, China). A two-cell probe design was used in this measuring technique. A single probe was used, in this design, to take measurements across the instrument’s whole dynamic range. The total conductivity of samples was determined by this method. The instrument was calibrated before going to sample analysis. The probe was then inserted into the sample, and the probe’s slot was completely immersed. To eliminate any bubbles that had entrapped in the slot, the sample was stirred for 5–10 s with the probe. The sample’s conductivity values were then displayed on the meter automatically [13]. In this way, the conductivity of all samples was measured.

### 2.9. Further Evaluation of the Selected Formulation

According to the above investigations, a suitable formula was selected and utilized for further evaluations to confirm its acceptance as the best formula.

#### 2.9.1. Acoustic Impedance

**Figure 2:**
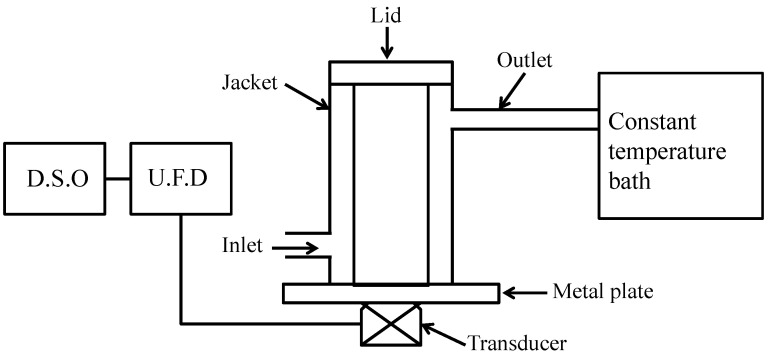
Schematic diagram of the experimental setup.

A unique experimental setup was designed to calculate the acoustic impedance of a selected gel formulation (S1) and a commercial gel (CG) at room temperature. Figure 2 displays a schematic diagram of the ultrasonic apparatus to illustrate the measuring technique. The device was fabricated by utilizing a pipe welded to an aluminum alloy plate that had been carefully washed and polished, as shown in Figure 2. Test samples were poured into a lidded aluminum alloy container. It was built to avoid air bubbles that might be produced while covering the samples in the container. The transmitter-receiver transducer, or probe, was attached beneath the aluminum plate by utilizing glue. The temperature of the samples was maintained by the flow of water from a thermostatically regulated water bath around the device’s close-fitting metal jacket. For all of the measurements, an ultrasonic instrument, the Sonatest UFD 300, Buckinghamshire, UK, was utilized to send and receive signals to and from the probe. On a Digital Storage Oscilloscope (DSO, Instek America Corp, Montclair, CA, USA) the received output from UFD was shown. The echo heights of the reflected ultrasound waves from the sample interface and the base aluminum plate were displayed on the DSO. A 2 MHz frequency probe was utilized throughout the experiment.

Acoustic impedance is a physical characteristic of tissue. It defines how much opposition an ultrasound beam experiences as it goes through a tissue. Mathematically, it is described as Z = ρv, where ρ is the density of the medium, and v is the speed of ultrasound in the medium. At the borderline between media of distinctive acoustic impedances, some of the wave energy is transmitted and some is reflected. The larger the gap in acoustic impedance between the two media, the more prominent will be the reflection, and the less there will be the transmission. For diagnostic ultrasound (US), ultrasound gel having acoustic impedance comparable to soft tissue (1.6 Mrayl) is necessary for the complete transmission of ultrasound waves.

When the acoustic impedances of both materials that make up the boundary are known, the reflection coefficient (fraction of the incident wave intensity that is reflected) can be calculated with the equation below.

(4) $$R=\frac{\left(Z_{1}-Z_{0}\right)}{\left(Z_{1\;}+Z_{0}\right)}$$

where, *Z*_1_ and *Z*_0_ are the impedances of the reflecting and incident medium, respectively. The Equation (5) gives the acoustic pressure of the first echo obtained from the first reception of a reflected wave at the interface of two media,

(5) $$P_{1}=KP_{0}e^{-2ad_{m}}\;R\;$$

The second echo’s acoustic pressure from the second reflected wave reception is expressed in the equation below;

(6) $$P_{2}=K^{2}P_{0}e^{-4ad_{m}}\;R^{2}r$$

Similarly, the acoustic pressure of the fourth echo, P_4_, can be expressed as

(7) $$P_{4}=K^{4}P_{0}e^{-8ad_{m}}\;R^{4}r^{3}$$

*K* is the constant dependent upon the interface between the base and the transducer, *P*_0_ is the acoustic pressure of the wave produced at the transducer, *d_m_* is the thickness of the base, *α* is the attenuation in the base material, *R* is the reflection coefficient at the base-medium (gel) interface, and *r* is the reflection coefficient for the base-transducer interface.

Equation (7) indicates that all parameters are independent of the tested gel samples above the acoustic interface surface except the parameter *R*, which depends upon the properties of the top tested gel medium interfacing with the aluminum plate. If *R*_1_ and *R*_2_ are the reflection coefficients from medium 1 and medium 2 above the base material, then Equation (7) can be reduced further as the ratio of acoustic pressures from the two media.

(8) $$\left[\frac{\left(5\right)P_{4\;}}{\left(6\right)P_{4}}\right]={\left[\frac{R_{1}}{R_{2}}\right]}^{4}$$

The transducer generated short ultrasound waves in this experiment; the acoustic pressure produced in the gel medium by the passing of these ultrasound pulses can be considered as proportional to the echo heights (signal amplitudes) seen on the oscilloscope display. Let *V*_1_ and *V*_2_ be the echo heights corresponding to the fourth echoes from medium 1 and medium 2. Equation (8) then modifies as follows

(9) $$\left[\frac{V_{1\;}}{V_{2}}\right]={\left[\frac{R_{1}}{R_{2}}\right]}^{4}$$

In terms of acoustic impedance, Equation (9) can be modified as

(10) $$\left[\frac{V_{1\;}}{V_{2}}\right]={\left[\frac{R_{1}}{R_{2}}\right]}^{4}={\left[\frac{Z_{1}-Z_{metal}}{Z_{1}+Z_{metal}}\times \frac{Z_{2}+Z_{metal}}{Z_{2}-Z_{metal}}\right]}^{4}$$

where, *Z*_1_, *Z*_2_, and *Z_metal_* are the acoustic impedances of the reflecting medium of fluid 1, fluid 2, and the acoustic impedance of metal, respectively. The value of *Z_meta_*_l_ can be calculated by using air and water as mediums 1 and 2.

(11) $$\left[\frac{V_{water\;}}{V_{air}}\right]={\left[\frac{Z_{water}-Z_{metal}}{Z_{water}+Z_{metal}}\times \frac{Z_{air}+Z_{metal}}{Z_{air}-Z_{metal}}\right]}^{4}$$

The literature value of *Z_water_* is 1.5 Mrayl, *Z_air_* is 0.0004 Mrayl, and *Z_meta_*_l_ is 20 Mrayl. In comparison to *Z_metal_*, *Z_air_* is negligible and hence the reflection coefficient of air [second term in Equation (11)] will be 1. In that situation, Equation (11) can be modified as:

(12) $$\left[\frac{V_{water\;}}{V_{air}}\right]={\left[\frac{Z_{metal}-Z_{water}}{Z_{metal}+Z_{water}}\right]}^{4}\mathrm{Or}Z_{metal}=Z_{water\;}\times \left(\frac{1+A}{1-A}\right)$$

where, A = ${\left(\frac{V_{water}}{V_{air}}\right)}^{1/4}$

Furthermore, medium 1 with a value of *Z_air_* and medium 2 with a value of *Z_Water_* are taken as reference standards for computing Z_gel_ impedance of both gels (S1, and CG) by adding the values of *Z_metal_* and *Z_gel_* in the Equation (12);

(13) $$\left[\frac{V_{water\;}}{V_{gel}}\right]={\left[\frac{Z_{metal}-Z_{water}}{Z_{water}+Z_{metal}}\right]}^{4}\times {\left[\frac{Z_{gel}+Z_{metal}}{Z_{metal}-Z_{gel}}\right]}^{4}$$

This implies:

(14) $$\left[\frac{V_{water\;}}{V_{gel}}\right]=\left[\frac{V_{water\;}}{V_{air}}\right]\times {\left[\frac{Z_{gel}+Z_{metal}}{Z_{metal}-Z_{gel}}\right]}^{4}$$

This is further simplified as:

(15) $$\left[\frac{V_{air\;}}{V_{gel}}\right]={\left[\frac{Z_{gel}+Z_{metal}}{Z_{metal}-Z_{gel}}\right]}^{4}$$

This can be further simplified as:

(16) $$B=\left[\frac{Z_{gel}+Z_{metal}}{Z_{metal}-Z_{gel}}\right]\mathrm{OR}Z_{gel}=Z_{metal}\times \left(\frac{B-1}{B+1}\right)$$

where, B = ${\left(\frac{V_{air}}{V_{gel}}\right)}^{1/4}$

Replacing the equation of Z_metal_ from Equation (12) in Equation (16)

(17) $$Z_{gel}=Z_{water}\frac{\left(1+A\right)}{\left(1-A\right)}\times \frac{\left(B-1\right)}{\left(B+1\right)}$$

Equation (17) gives the values of the acoustic impedance of both gels (selected formulation S1, and commercial gel CG) in terms of the acoustic impedance of water, peak to peak voltage from echoes with water, air, and ultrasound gel medium [41].

#### 2.9.2. Accelerated Stability Test

The accelerated stability test for a selected gel formulation was performed for a stable formulation by incubating it in an airtight bottle for 7 days at 70 °C temperature and 75% humidity. The suitable parameters such as color, pH, viscosity, and conductivity were also evaluated before and after the incubation period [13,42,43].

#### 2.9.3. Evaluation of Images Produced

This study was conducted at Abdullah Medical Complex(Faisalabad, Pakistan) in July 2021. The patient’s right kidney was examined by a sonographer for two kinds of ultrasound gel (commercial gel and the selected formulation). A curved transducer or probe, 4.5 MHz frequency, and an ultrasonographic machine (Mindray, model no. Z5, Thiruverkadu, Chennai, Tamil Nadu) were utilized for this study. The right kidney of the patient was imaged from the 12th intercostal space at the right side. The patient was observed thrice, first without ultrasound gel or any conductive medium, then utilizing commercial gel, and then, after 5 min, the examined part was cleaned with tissue paper and re-examined with the selected formulation. The quality of the image was inspected visually by the sonographer.

#### 2.9.4. Cost

The cost of the selected gel formulation was calculated and compared with the commercial gel.

## 3. Results and Discussions

### 3.1. Visual Inspection

**Figure 3:**
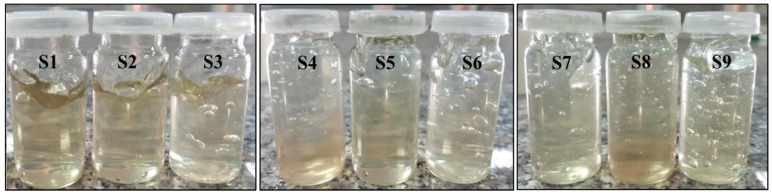
Visual inspection of all samples.

All the formulations were transparent and clear due to the property of carbopol 980 polymer to produce clear gels. Their texture and appearance were also smooth. All the formulations showed a slight green color due to the presence of pure natural aloe vera gel, as shown in Figure 3.

### 3.2. Homogeneity

The end results revealed that all of the formulations were homogenous with no evidence of the presence of any type of smear. Their appearance, touch, feel, and the odor were all acceptable. All of the formulations were non-greasy, and they could be readily removed by rubbing the gel on the skin with tissue paper.

### 3.3. VIS-Spectrophotometry

**Figure 4:**
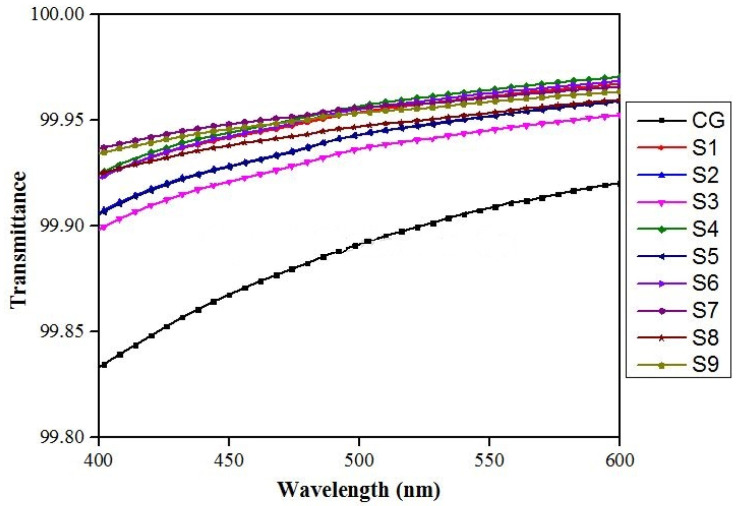
Transmittance of all formulations (S1, S2, S3, …, S9), along with commercial gel (CG) in the visible range.

All the formulations (S1, S2, S3, …, S9) showed excellent transmittance in the visible region, as shown in Figure 4. So, we can conclude that all formulations were visibly clear and had excellent transparency.

### 3.4. pH

**Figure 5:**
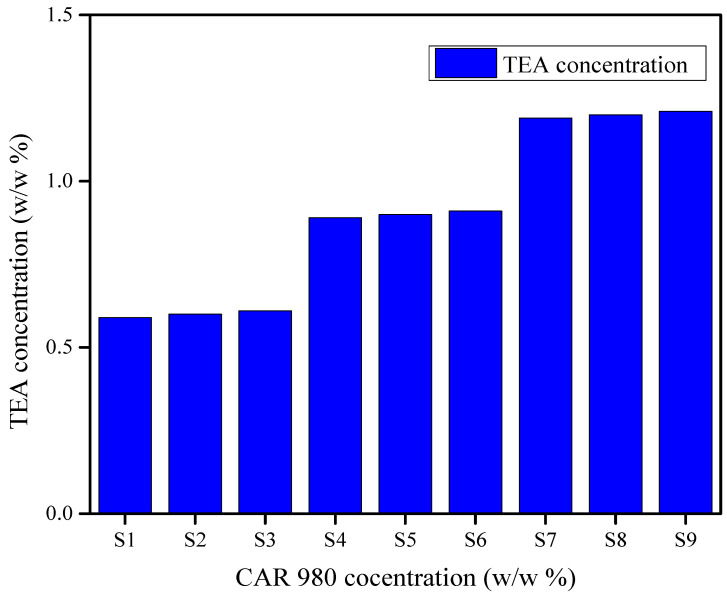
An increase in the concentration of triethanolamine was observed with an increase in the polymer (CAR 980) concentration to attain the pH of all formulations between 7 and 7.4.

The pH of all formulations was between 7 and 7.4. Triethanolamine was used to adjust the pH of prepared formulations and there was an increase in the concentration of triethanolamine used was observed as the concentration of polymer (CAR 980) increased from S1 to S9. The utilization of triethanolamine in relation to CAR 980 polymer concentration to attain the pH of all formulations between 7 and 7.4 is shown in Figure 5.

### 3.5. Skin Irritation Test

**Figure 6:**
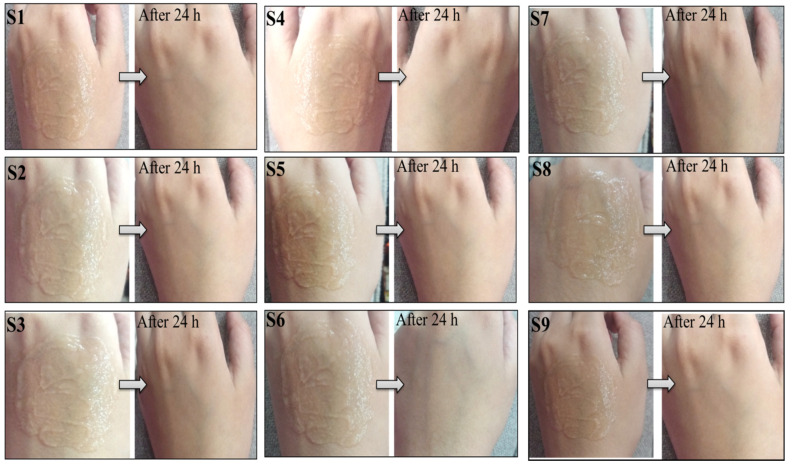
During the study period of 24 h, no redness, edema, irritation, or any other negative effect was observed.

During the irritancy test, the formulations did not cause irritation, edema, redness, or any other negative effects on the skin, as shown in Figure 6. As a result, it was concluded that all formulations were safe to use for external applications as they had a proper pH compatible with skin secretions. It was also due to the selection of ingredients that have no harmful effects on the skin, as well as the presence of glycerine and aloe vera gel, which provide skin conditioning and anti-inflammatory properties to the gel.

### 3.6. Viscosity Determination

**Figure 7:**
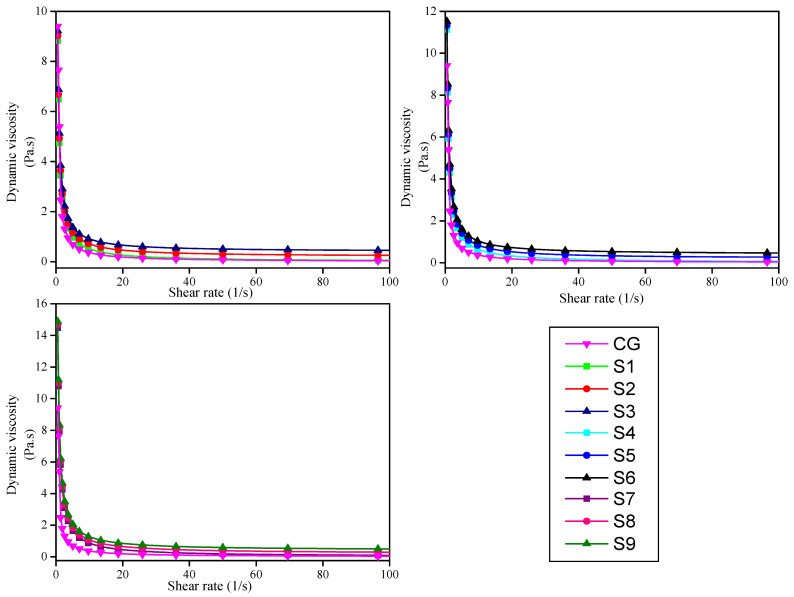
Dynamic viscosity (Pa.s) of all formulations when compared to commercial gel at different shear rates (1/s).

A gel’s viscosity is a measurement of its resistance to flow, and the consistency of any gel formulation is determined by its viscosity. The viscosity of ultrasound gel is important since it is difficult to maintain a localized geographic region on the skin with a lower viscosity of gel, especially when the ultrasound procedure is performed in a vertical body position. The commercial gel showed its maximum viscosity of 9.4 Pa.s at 0.5 (1/s) shear rate and decreases smoothly to near zero as the shear rate increases, as shown in Figure 7. The S9 formulation containing 0.80% concentration of carbopol 980 polymer showed the highest viscosity as compared to the S1 formulation containing 0.40% concentration, which showed the lowest viscosity of all samples at 30 °C temperature and 0.5 (1/s) shear rate. The viscosity of formulations increases as the concentration of polymer increases, as shown in Figure 7. But the viscosity of all samples decreases as the shear rate of geometry increases and drops to near zero. In other words, we can say that viscosity shows a positive linear response to polymer concentration and a negative linear response to shear rate. The one formulation with 0.40% polymer concentration (S3) showed almost similar viscosity as a commercial gel at 0.5 (1/s) shear rate and 30 °C temperature.

### 3.7. Antibacterial Activity

**Figure 8:**
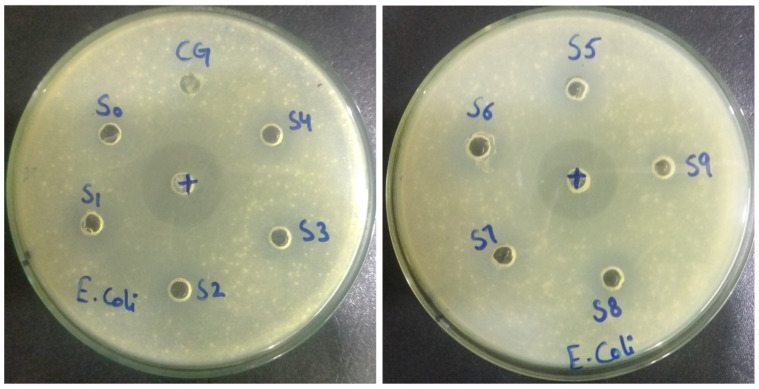
Nutrient agar plates showing zones of inhibition of all formulations along with commercial gel (CG), blank sample (S0), and control antibacterial Ciprofloxacin (+) against Gram-negative bacteria (*Escherichia coli*) after an incubation period of 24 h at 37 °C.

A clear zone was formed around the samples, showing that the bacterial growth was inhibited by all the formulations and commercial gel, as shown in Figure 8. The zone of inhibition (in millimeters) of all formulations is shown in Table 1. The zone of inhibition (circle around the samples) showed that the prepared formulations represent a larger inhibition zone than commercial gel but a smaller inhibition zone than the control antibacterial Ciprofloxacin. The antibacterial activity of the blank sample (S0) was also observed, possibly due to the presence of a chelating agent (Disodium EDTA). The S1 formulation showed slightly higher antibacterial activity than commercial gel.

### 3.8. Conductivity

Ultrasound gel generally functions as a conductive medium, and information regarding the quality of images is given by the conductivity results. The conductivity values of all samples are shown in Table 2. Due to the presence of pure natural aloe vera gel, the conductivity values of the prepared formulations were found to be greater than those of the commercial gel. The higher conductivity of prepared formulations did not cause any problem in the ultrasonography procedure.

### 3.9. Further Evaluation of the Selected Formulation

The ultrasound gel formulation with 0.40% carbopol and 0.20% methylparaben concentration (named S1) was selected for further evaluation due to its high transparency, clarity, homogeneity, good antibacterial activity, and almost similar viscosity as compared to commercial gel.

#### 3.9.1. Acoustic Impedance

The acoustic impedance of the selected formulation (S1) was 1.45 Mrayl and the acoustic impedance of commercial gel (CG) was 1.5 Mrayl. The results showed that the acoustic impedance of both was very close and similar to the impedance of soft tissues (1.6 Mrayl). The intensity reflection coefficient tells us that 0.6% of sound waves reflect at the boundary between gel and human skin, while 99.4% transmit through the skin, and it is opposite without ultrasound gel.

#### 3.9.2. Accelerated Stability Test

The selected formulation (S1) was stable over the whole incubation period and showed almost similar properties before and after the incubation period, as shown in Table 3. So, we can conclude that the formulated ultrasound gel has a shelf life of one year.

#### 3.9.3. Evaluation of Images Produced

**Figure 9:**
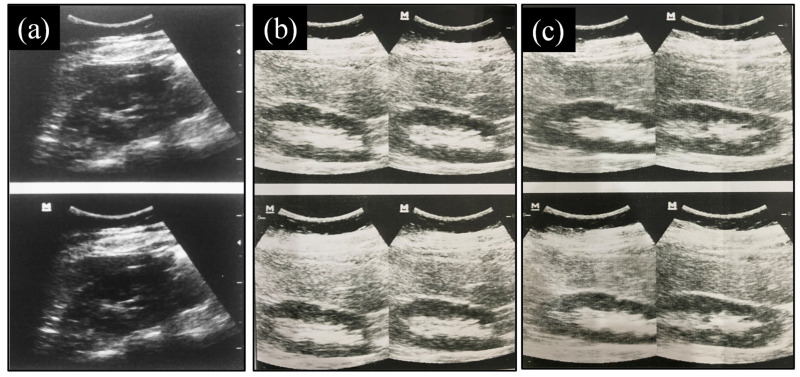
Ultrasound image of the right kidney produced (**a**) without any conductive medium, (**b**) when a selected formulation (S1) was applied to the skin, and (**c**) when a commercial gel (CG) was applied to the skin.

There was no difference between the texture, feel, spreadability, and transparency of the selected formulation (S1) and commercial gel (CG) observed by the sonographer, except for viscosity, which was slightly lower. In addition, the patient did not feel any discomfort, itching, or any other skin problem during ultrasonography. The good and clear image quality of the right kidney was achieved from the ultrasonogram when using the selected ultrasound gel formulation (S1). The ultrasound image produced without any medium was very blurred and did not give any information about the organ. The overall results suggested that there was no distinction between the image quality produced by commercial gel and the selected formulation (S1), as shown in Figure 9. This demonstrated that the gel was suitable for ultrasonography because no further issues were encountered.

#### 3.9.4. Cost

The cost of the selected formulation (S1) was 5.5 $ /kg, while the cost of commercial gel (Aquasonic 100) was 19.99 $ /kg. The complete cost analysis of the selected formulation (S1) is shown in Table 4. In this analysis, the manufacturing or electricity, and packaging costs of formulated ultrasound gel were not included.

## 4. Conclusions

Ultrasonography is a non-invasive technique for assessing and diagnosing a wide variety of medical conditions related to internal organs. It utilized ultrasound gel as a conductive medium for transmitting ultrasound waves between the transducer and skin. In this study, an alternative ultrasound gel formulation was prepared by using biodegradable and natural ingredients such as aloe vera gel. Aloe vera gel has an anti-inflammatory effect on the skin. Aloe vera gel is inexpensive and commonly available too. The formulation prepared with it is safe for patients with sensitive skin due to its hypoallergenic characteristics. The S1 formulation, among all, showed similar properties to commercial gels. This formulation had a proper pH of 7 ± 0.4, indicating that it is compatible with skin secretions. Stability parameters like viscosity, visual appearance, and antibacterial activity of the formulation (S1) showed that there was no significant change observed during the study period. The overall results suggested that there was no distinction between the image quality produced by commercial gel (CG) and the selected formulation (S1) when applied to the skin. So, this formulation could be used to take medical images during ultrasound scans with no harmful effect on the skin and is considered a successful alternative to commercial gel. In the future, the formulation will be further improved to commercial standards and tested with a large number of patients to confirm its efficiency, as, in this research, only one organ of a healthy patient was imaged.

## 5. Materials and Methods

### 5.1. Materials

Materials included Carbopol 980 (CAR 980, >99% purity), Methylparaben (99% purity), Propylparaben (>99% purity), Disodium ethylenediaminetetraacetic acid (Disodium EDTA, 99% purity), Glycerine (99.5% purity), and Triethanolamine (TEA, ≥99% purity) were purchased from Sigma-Aldrich, Gillingham, UK. The natural ingredient required for the formulation of ultrasound gel was aloe vera gel, which was collected from the aloe vera plant grown in the home garden.

### 5.2. Method

#### 5.2.1. Preparation of Aloe Vera Gel

**Figure 10:**
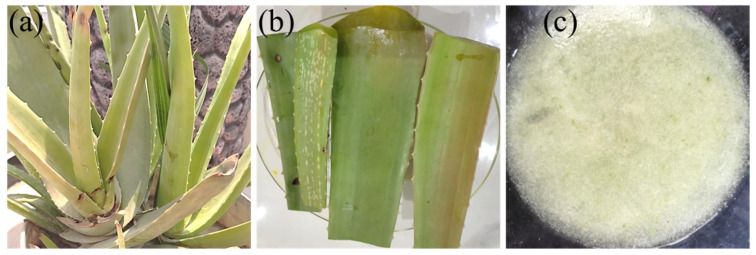
(**a**) Aloe vera plant, *Aloe barbadensis*, (**b**) washed aloe vera leaves, and (**c**) natural aloe vera gel.

Freshly collected leaves from the aloe vera plant (*Aloe barbadensis*) were washed thoroughly with distilled water to remove the yellow fluid secretion and residues, shown in Figure 10. Then the epidermis of the aloe vera leaves was peeled off and the aloe vera gel (parenchymatous tissue) was collected and ground into liquid form using a mixer grinder. Then, the gel was filtered to remove any remaining particulates in the liquid. The freshly prepared aloe vera gel was covered with aluminum foil, to prevent contamination, and kept in the refrigerator for further use [22].

#### 5.2.2. Preparation of Ultrasound Gel Formulations

**Figure 11:**
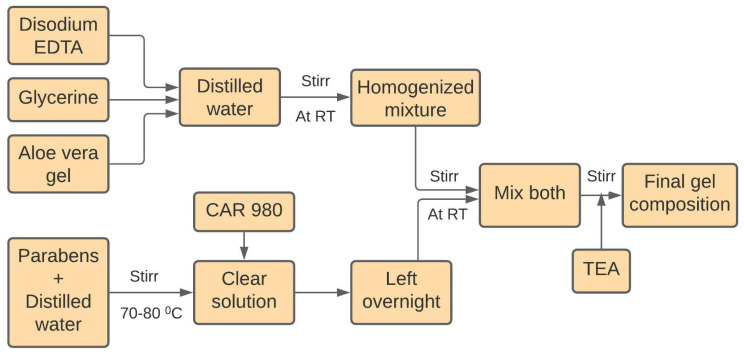
Flowchart for the synthesis of carbopol based ultrasound gel formulations.

Nine gel formulations containing three different concentrations of the polymer carbopol 980 (0.4%, 0.6%, and 0.8%) and methylparaben (0.2%, 0.3%, and 0.4%) with other ingredients were prepared, as shown in Table 5. First of all, accurately weighed amounts of methylparaben and propylparaben were dissolved in distilled water with constant stirring by using a magnetic stirrer at 70–80 °C. Then carbopol 980 polymer was dispersed in it. Final dispersion was left overnight for complete swelling of the polymer. Secondly, a weighed quantity of disodium EDTA was dissolved in a small quantity of distilled water, and then aloe vera gel and glycerin were added under constant stirring. The next day, both mixtures were mixed with continuous stirring until homogenous. Finally, pH was adjusted to 7 with triethanolamine solution at room temperature with constant mixing by a mechanical stirrer to achieve the final gel composition [14]. In the final step, the gel was transferred into different squeeze bottles and stored at the proper temperature for further use. The complete synthesis process is shown in Figure 11.

## Figures and Tables

**Table 1:** Inhibition zone measurement for antibacterial activity.

Samples	Inhibition Zone (mm) against *Escherichia Coli*
Control antibacterial (Ciprofloxacin)	32
CG (Commercial Gel)	15
S0	10
S1	18
S2	23
S3	26
S4	21
S5	24
S6	26.5
S7	21
S8	24.5
S9	27

**Table 2:** Conductivity values of prepared ultrasound gel formulations and commercial ultrasound gel.

Samples	Conductivity Values at 28 °C
CG (commercial gel)	0.70 mS/cm
S1	2.10 mS/cm
S2	2.14 mS/cm
S3	2.12 mS/cm
S4	2.27 mS/cm
S5	2.25 mS/cm
S6	2.20 mS/cm
S7	2.50 mS/cm
S8	2.48 mS/cm
S9	2.52 mS/cm

**Table 3:** Different characteristics of the selected formulation (S1) before and after the incubation period.

Characteristics	Before Incubation	After Incubation
pH	7	7
Color	Slight green	Slight green
Transparency	Yes	Yes
Viscosity	8.92 Pa.s	8.82 Pa.s
Antimicrobial activity	Yes	Yes
Conductivity	2.10 mS/cm	2.12 mS/cm

**Table 4:** Cost analysis of the selected formulation (S1) in comparison with the commercial gel.

Materials	Price Per kg ($)	*w*/*w*% of Materials Required Per kg	Cost ($)	Total Cost of S1 Per kg	Cost of Commercial Gel (Aquasonic 100) Per kg
CAR 980	19	4	0.07	5.5 $	19.99 $
Methylparaben	928	2	1.85		
Propylparaben	217	0.5	0.10		
Disodium EDTA	427	0.2	0.08		
Glycerine	2	40	0.08		
Distilled water	3	≈900	2.7		
Aloe vera gel	0.5	50	0.02		
TEA	76	≈8.62	0.65		

**Table 5:** Composition of different gel formulations, each concentration is based on *w*/*w*% of the total sample (100 g).

Samples	Carbopol	Methylparaben	Distill. Water	Propylparaben	Disodium EDTA	Glycerine	Aloe Vera Gel
S1	0.4	0.2	Remaining	0.05	0.02	4	5
S2		0.3					
S3		0.4					
S4	0.6	0.2					
S5		0.3					
S6		0.4					
S7	0.8	0.2					
S8		0.3					
S9		0.4					

## Data Availability

Not applicable.
